# MicroRNA-27 inhibits adipogenic differentiation in orbital fibroblasts from patients with Graves’ orbitopathy

**DOI:** 10.1371/journal.pone.0221077

**Published:** 2019-08-15

**Authors:** Sun Young Jang, Min Kyung Chae, Joon H. Lee, Eun Jig Lee, Jin Sook Yoon

**Affiliations:** 1 Department of Ophthalmology, Soonchunhyang University Bucheon Hospital, Soonchunhyang University College of Medicine, Bucheon, Republic of Korea; 2 Department of Ophthalmology, Severance Hospital, The Institute of Vision Research, Yonsei University College of Medicine, Seoul, Republic of Korea; 3 Myung-Gok Eye Research Institute, Konyang University College of Medicine, Seoul, Republic of Korea; 4 Department of Endocrinology, Severance Hospital, Institute of Endocrine Research, Yonsei University College of Medicine, Seoul, Republic of Korea; Case Western Reserve University, UNITED STATES

## Abstract

**Background:**

To investigate the role of microRNA (miR)-27a and miR-27b in adipogenesis in an *in vitro* model of Graves’ orbitopathy (GO).

**Methods:**

Orbital fat tissues were harvested from GO and non-GO participants for primary orbital fibroblast cultures. The expression levels of miR-27a and miR-27b between GO and non-GO orbital fat tissues were compared. During adipogenesis of GO orbital fibroblasts, the expression levels of miR-27a and miR-27b were determined, and the effects of mimics of miR-27a and miR-27b transfection on adipogenesis of GO orbital fibroblast were investigated.

**Results:**

Real time-polymerase chain reaction showed significantly more decreases in miR-27a and miR-27b levels in orbital fat tissues in GO participants than in non-GO participants (p < 0.05). The expression of both miR-27a and miR-27b was highest in orbital fibroblasts at day 0 and declined gradually after the induction of adipogenic differentiation. The expression levels of PPARγ, CCAAT/enhancer binding protein (C/EBP)α and C/EBPβ were decreased and Oil Red O-stained lipid droplets were lower in GO orbital fibroblasts transfected with miR-27a and miR-27b mimics than in negative controls.

**Conclusions:**

Our results indicated that miR-27a and miR-27b inhibited adipogenesis in orbital fibroblasts from GO patients. Further studies are required to examine the potential of miR-27a and miR-27b as targets for therapeutic strategies.

## Introduction

Graves’ orbitopathy (GO) is an inflammatory autoimmune disorder of the orbit [1–3]. The clinical manifestations of GO reflect an increase in orbital volume, which might lead to proptosis, lid swelling, corneal exposure, diplopia, and compressive optic neuropathy [2–5]. Patients with GO often suffer more from a disfiguring appearance than from their general condition due to Graves’ disease.

Adipogenesis, the transition of activated orbital fibroblasts into adipocytes, can lead to orbital proptosis in GO patients [6]. Proptosis, which is a leading cause of a disfiguring appearance, is usually treated with orbital decompression [7–9]. Even though orbital decompression is a well-established procedure for treating proptosis, the risks of postoperative complications such as infection, bleeding, surgically-induced diplopia, or insufficient proptosis reduction still exist [10–12]. There are therefore continuous efforts to identify methods to block the adipogenesis pathway as a new therapeutic option.

MicroRNAs (miRs) are non-coding single-stranded RNAs, comprised of approximately 22 nucleotides, which exert post-transcriptional effects on gene expression. There is growing evidence for the role of miRNAs in regulating the pathway of adipogenesis, and several miRNAs have been found to be involved in adipogenesis [13]. MiR-27 is one of a number of miRs implicated in regulating cholesterol and fatty acid metabolism [14]. The miR-27 gene family has been shown to be downregulated during the differentiation of adipocytes. MiR-27 inhibits adipocyte formation when overexpressed by blocking the expression of peroxisome-proliferator activated receptor-γ (PPARγ) and CCAAT/enhancer binding protein (C/EBP)α [15].

In GO patients, adipogenesis is one of the most important pathomechanisms. We previously used microarray analysis to identify miRNAs involved in the pathogenesis of GO [16]. We found that 43 miRNAs, which included miR-27a, were dysregulated in orbital tissue from GO patients when compared to healthy controls. Based on this background, in the present study, we investigated the roles of miR-27a and miR-27b in adipogenesis using an *in vitro* model of GO.

## Materials and methods

### Subjects and cell culture/adipogenesis protocols

Orbital adipose tissue was obtained from 13 GO patients and 8 non-GO subjects. All GO patients satisfied the following conditions: 1) patients underwent orbital decompression for proptosis correction; 2) euthyroid and inactive GO status at the time of surgery; and 3) patients had not been treated with steroids or radiation therapy for at least 3 months. All control subjects satisfied the following conditions: 1) age- and sex-matched to GO subjects; 2) without thyroid or any other inflammatory diseases; and 3) control subjects underwent cosmetic upper and lower blepharoplasty. Informed written consent was obtained from all participants, and this study was approved by the Institutional Review Board of the Severance Hospital, Yonsei University College of Medicine.

To compare the expression levels of miR-27a and miR-2b between GO and non-GO tissues, orbital adipose/connective tissue explants from five GO patients and five control subjects were used.

Orbital tissues were obtained from eight GO patients and three non-GO patients to perform cell-based experiments. Orbital fibroblast cell cultures were performed according to methods described previously [16–19]. Procedures for adipogenesis and lipid droplet examination were performed as described previously [18,20].

All experiments were independently performed at least three times using at least three cell cultures harvested from different individuals. The results are presented as the mean ± standard deviation. Differences between groups were assessed by independent and paired *t-*tests. In all analyses, p < 0.05 was assumed to indicate statistical significance.

### Cell transfection with miR-27a and miR-27b mimics

Orbital fibroblasts were transfected with miR-27a and miR-27b mimics and each control according to the manufacturer’s protocols. The miR-27a and miR-27b mimics were obtained from Ambion/Applied Biosystems (Foster City, CA, USA). Cells were transfected with 50 nM of miR-27a and miR-27b mimics using commercial reagents (Lipofectamine RNAiMAX; Life Technologies, Carlsbad, CA, USA).

### Quantitative real time-polymerase chain reaction (qRT-PCR) and western blotting

qRT-PCR and western blotting were performed as described previously [16,19]. Briefly, for qRT-PCR, total RNA was extracted from orbital fibroblasts according to the manufacturer’s instructions. One μg of RNA was used to generate cDNA using a TaqMan miRs Reverse Transcription Kit (Applied Biosystems). The cDNA was amplified using a thermocycler (ABI StepOnePlus RT-PCR; Applied Biosystems) with TaqMan universal PCR master mix (No AmpErase UNG; Applied Biosystems). RNU6B expression was used for normalization. All PCRs were performed in triplicate. The results are presented as relative fold changes of threshold cycle (Ct) relative to the control group, determined using the 2^−ΔΔCt^ method.

The effects of miR-27a and miR-27b mimics on PPARγ, C/EBPα, and C/EBPβ protein release in GO orbital fibroblasts were analysed by western blotting. Transfected cells were washed with ice-cold phosphate-buffered saline, and whole-cell lysates were obtained by incubation on ice for 30 min in cell lysis buffer (20 mM HEPES, pH 7.2, 10% [v/v] glycerol, 10 mM Na_3_VO_4_, 50 mM NaF, 1 mM phenylmethylsulphonyl fluoride, 0.1 mM dithiothreitol, 1 μg/mL leupeptin, 1 μg/mL pepstatin, and 1% [v/v] Triton X-100). Reagents were purchased from Sigma-Aldrich (St. Louis, MO, USA). Lysates were centrifuged at 12,000 × *g* for 10 min and the cell homogenate fractions were stored at –70°C until use.

Protein concentrations were determined by the Bradford assay. Equal amounts of protein (50 μg) were boiled in sample buffer and resolved by 10% (w/v) sodium dodecyl sulfate-polyacrylamide gel electrophoresis. Proteins were transferred onto polyvinylidene difluoride membranes (Immobilon; Millipore, Bedford, MA, USA). The samples were incubated overnight with primary antibodies (PPARγ, C/EBPα, and C/EBPβ) in Tris-buffered saline with Tween 20 (TBST), and washed three times with TBST. Immunoreactive bands were detected with horseradish peroxidase-conjugated secondary antibody, signal developed using an enhanced chemiluminescence kit (Amersham Pharmacia Biotechnology, Inc., Piscataway, NJ, USA) and detected by exposure to X-ray film (Amersham Pharmacia Biotechnology, Inc.). The immunoreactive bands were quantified by densitometry and normalized relative to the β-actin levels in the same sample.

### Oil Red O staining

Orbital fibroblast cultures were plated in a one-well culture disc in Dulbecco’s Modified Eagle’s Medium containing 10% foetal bovine serum, grown to confluence, and subjected to the differentiation protocol. The cells were washed twice with 1× phosphate-buffered saline, fixed in 10% formalin overnight at room temperature, and rinsed in 60% isopropanol before staining with filtered 0.21% Oil Red O in isopropanol-water for 1 h. Washed cells were exposed to Mayer’s haematoxylin solution (Sigma-Aldrich) for 5 min and rinsed with tap water before being visualized and photographed at 200× using a BX60 light microscope (Olympus, Melville, NY, USA).

To quantitatively evaluate of Oil Red O staining, cell-bound Oil Red O was solubilized with 100% isopropanol and the optical density of the solution was measured by a spectrophotometer at 490 nm.

## Results

### Comparison of miR-27a and miR-27b expression in GO and non-GO participants

**Fig 1:**
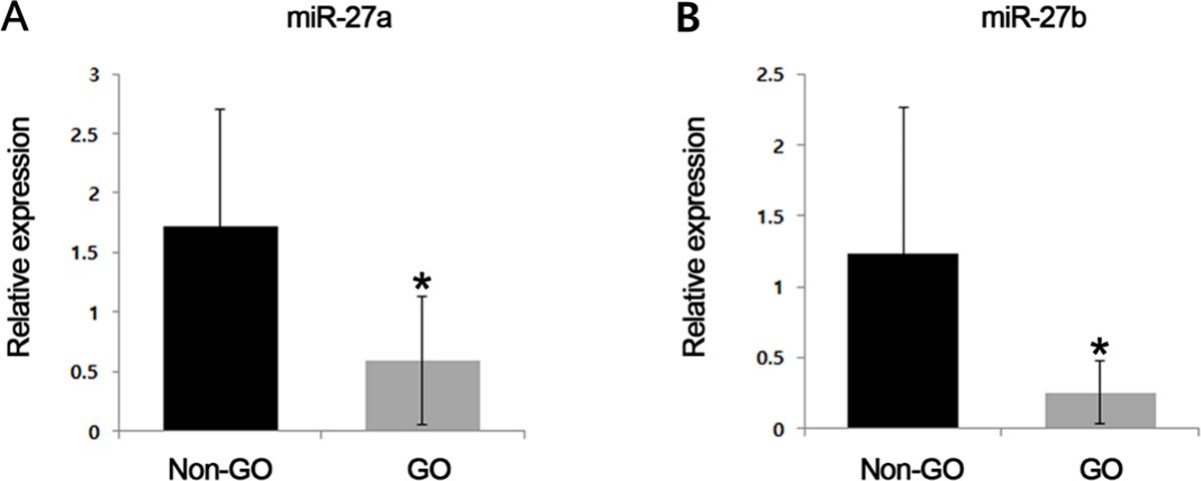
Comparison of microRNA-27 (miR-27)a and miR-27b expression in Graves’ orbitopathy (GO) and non-GO control participants. (A) The expression levels of miR-27a and (B) miR-27b were significantly lower in orbital adipose tissues from GO than from non-GO participants (*P<0.05).

The miR-27a and miR-27b expression levels were determined in orbital fat tissue obtained from five GO and five non-GO participants using qRT-PCR. The expression levels of miR-27a and miR-27b were significantly lower in orbital adipose tissues from GO than from non-GO participants (p = 0.038 and p = 0.047, respectively; independent *t*-test; Fig 1).

### MiR-27a and miR-27b expressions during adipogenesis in GO orbital fibroblasts

**Fig 2:**
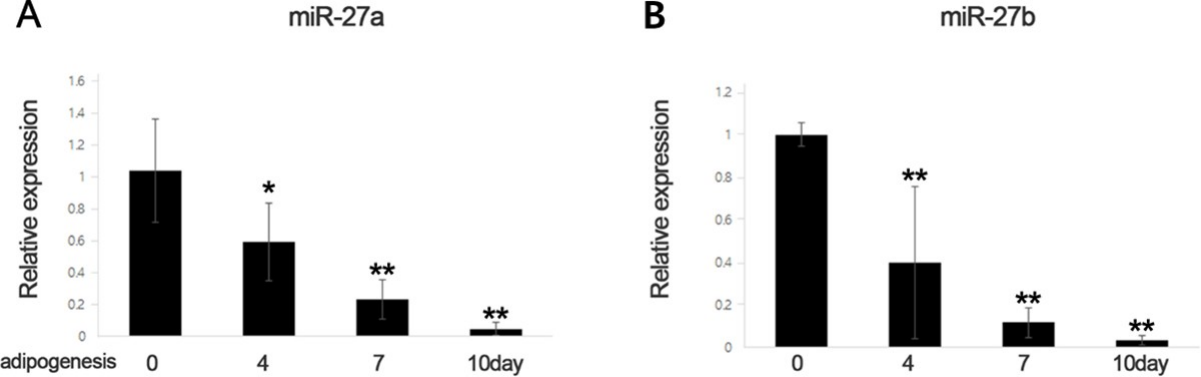
MicroRNA-27 (miR-27)a and miR-27b expression during adipogenesis in Graves’ orbitopathy orbital fibroblasts. (A) The levels of miR-27a and (B) miR-27b were gradually decreased upon adipogenic differentiation. (*P<0.05, **P<0.01).

Changes in expression levels of miR-27a and miR-27b in GO orbital fibroblasts during adipogenesis were investigated using qRT-PCR. MiR-27a expression was highest in orbital fibroblasts at day 0 and declined gradually after the induction of differentiation (day 4, p = 0.049; day 7, p = 0.007; and day 10, p = 0.003; paired t-test compared to day 0) (Fig 2A). MiR-27b expression was also highest in orbital fibroblasts at day 0 and declined gradually after the induction of differentiation (day 4, p = 0.002; day 7, p < 0.001; and day 10, p < 0.001; paired t-test compared to day 0) (Fig 2B).

### Effects of miR-27a and miR-27b mimics on adipogenesis in GO and non-GO orbital fibroblasts

**Fig 3:**
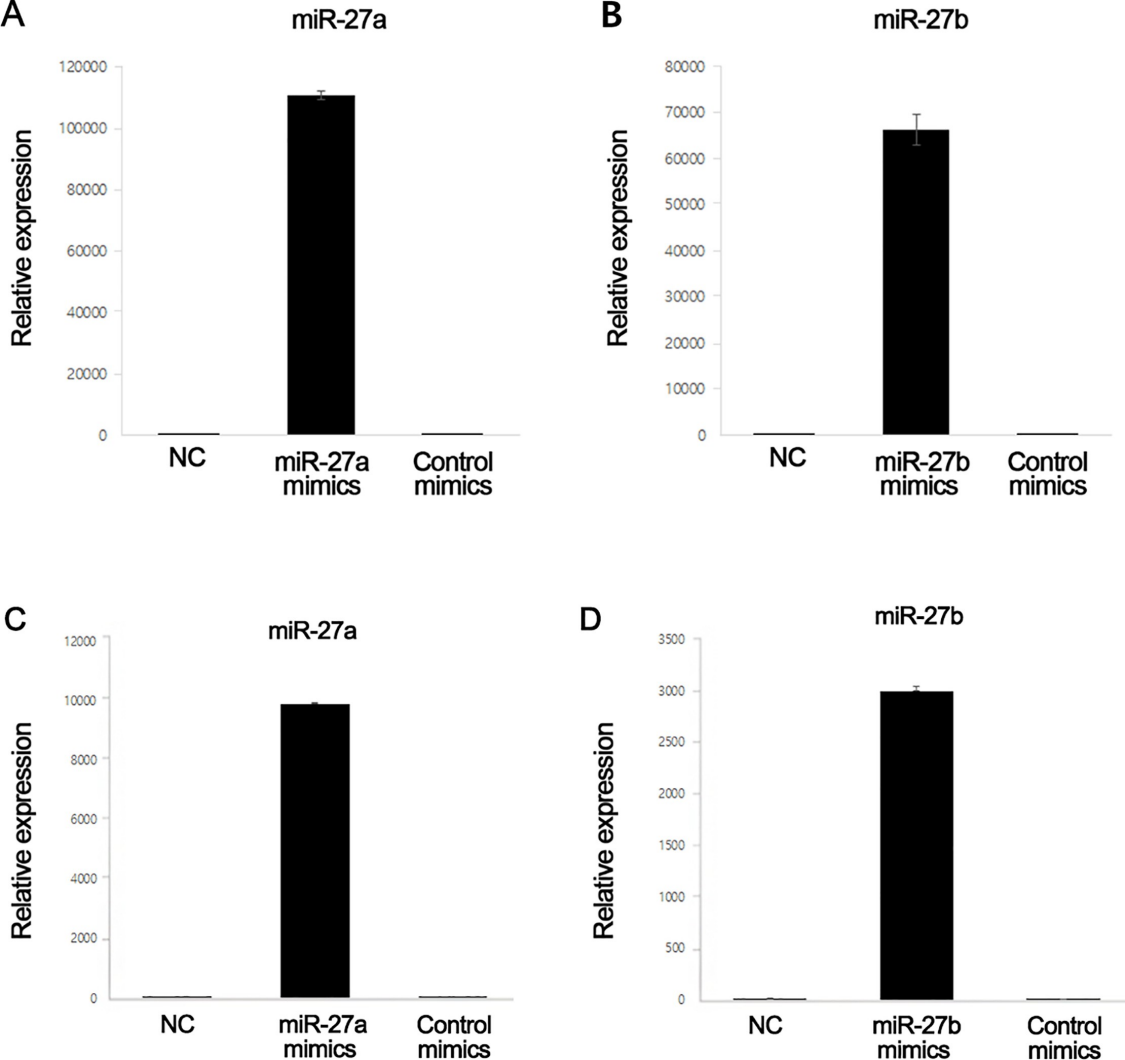
Cellular microRNA-27 (miR-27)a and miR-27b levels. (A) The levels of miR-27a and (B) miR-27b increased following transfection of miR-27a and miR-27b mimics in GO orbital fibroblasts. (C) The levels of miR-27a and (D) miR-27b increased following transfection of miR-27a and miR-27b mimics in non-GO orbital fibroblasts. NC, negative control.

The cellular miR-27a and miR-27b levels increased approximately 110,000- and 60,000-fold following transfection of miR-27a and miR-27b mimics in GO orbital fibroblasts, respectively (Fig 3A and 3B). The miR-27a and miR-27b levels also increased, by approximately 10,000- and 3,000-fold following transfection of miR-27a and miR-27b mimics in non-GO orbital fibroblasts, respectively (Fig 3C and 3D).

**Fig 4:**
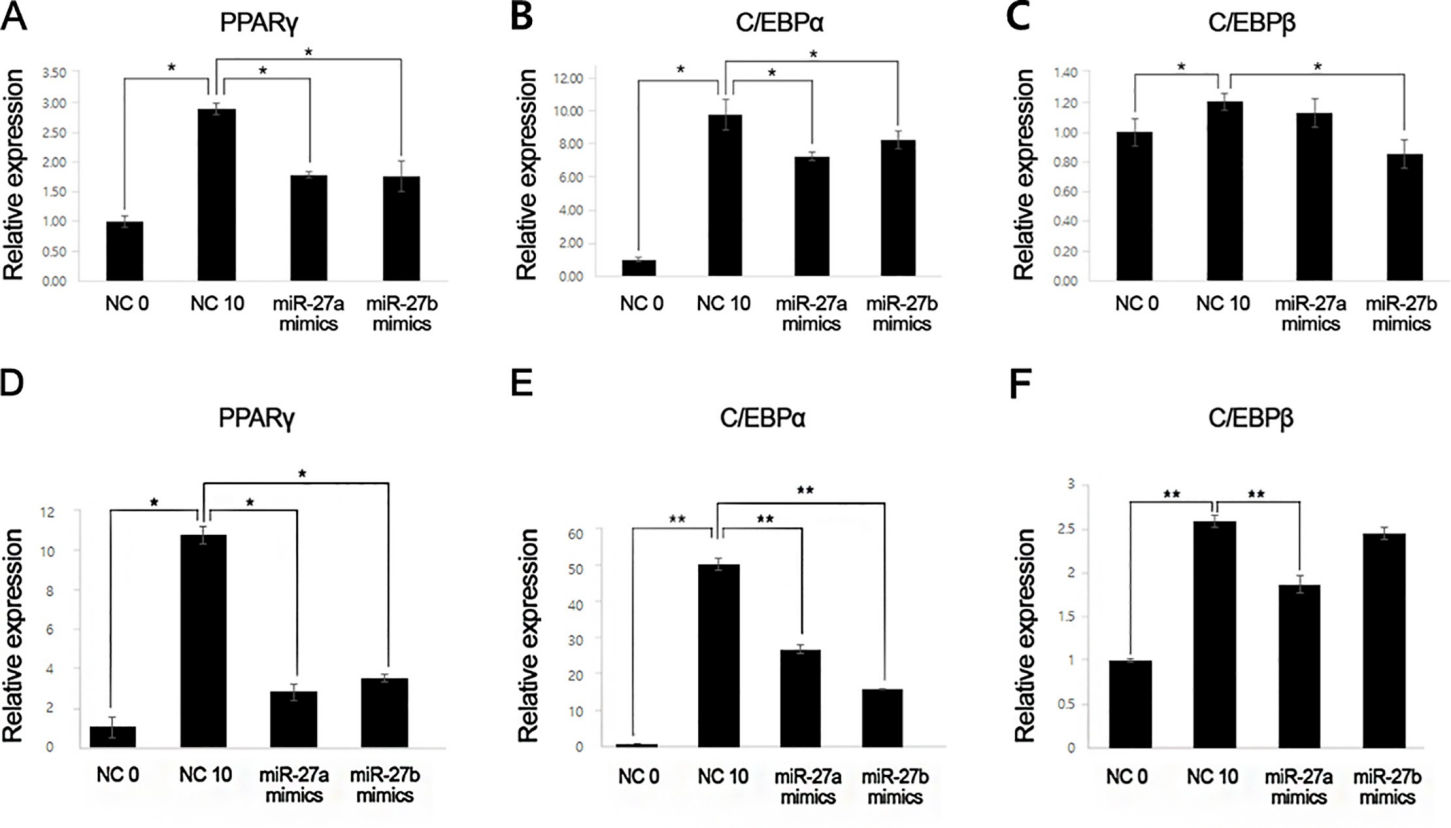
Effects of microRNA-27 (miR-27)a and miR-27b mimics on peroxisome-proliferator activated receptor-γ (PPARγ), CCAAT/enhancer binding protein (C/EBP)α, and C/EBPβ mRNA expression in Graves’ orbitopathy (GO) and non-GO orbital fibroblasts. (A, B) mRNA levels of PPARγ and C/EBPα were increased on day 10 of adipogenesis, and significantly decreased in GO orbital fibroblasts transfected with miR-27a and miR-27b mimics. (C) mRNA levels of C/EBPβ were significantly decreased in GO orbital fibroblast transfected with miR-27b mimics. The mRNA levels of C/EBPβ were decreased in GO orbital fibroblasts transfected with miR-27a mimics, albeit not significantly. (D, E) The mRNA levels of PPARγ and C/EBPα, were increased on 10 day of adipogenesis, and significantly decreased in orbital fibroblasts transfected with miR-27a and miR-27b mimics. (F) The mRNA levels of C/EBPβ were significantly decreased in orbital fibroblasts transfected with miR-27a mimics. The mRNA levels of C/EBPβ were decreased in GO orbital fibroblast transfected with miR-27b mimics, although not significantly. NC, negative control. (*P<0.05, **P<0.01).

To investigate changes in the mRNA levels of PPARγ, C/EBPα, and C/EBPβ, qRT-PCR was performed in GO and non-GO orbital fibroblasts. The mRNA levels of PPARγ were significantly decreased in GO orbital fibroblasts transfected with miR-27a and miR-27b mimics (both, p < 0.001). The mRNA levels of C/EBPα were also significantly decreased in GO orbital fibroblasts transfected with miR-27a and miR-27b mimics (p = 0.01 and p = 0.03, respectively). The mRNA levels of C/EBPβ were decreased in GO orbital fibroblasts transfected with miR-27a and miR-27b mimics (p = 0.15 and p = 0.001, respectively) (Fig 4A–4C).

We found similar trends in the mRNA levels of PPARγ, C/EBPα, and C/EBPβ in non-GO orbital fibroblasts (Fig 4D–4F). The mRNA levels of PPARγ were significantly decreased in GO orbital fibroblasts transfected with miR-27a and miR-27b mimics (p = 0.03 and p = 0.04, respectively). The mRNA levels of C/EBPα were also significantly decreased in GO orbital fibroblasts transfected with miR-27a and miR-27b mimics (both, p<0.01). The mRNA levels of C/EBPβ were decreased in GO orbital fibroblasts transfected with miR-27a and miR-27b mimics (p<0.01 and p = 0.08, respectively) (Fig 4D–4F).

**Fig 5:**
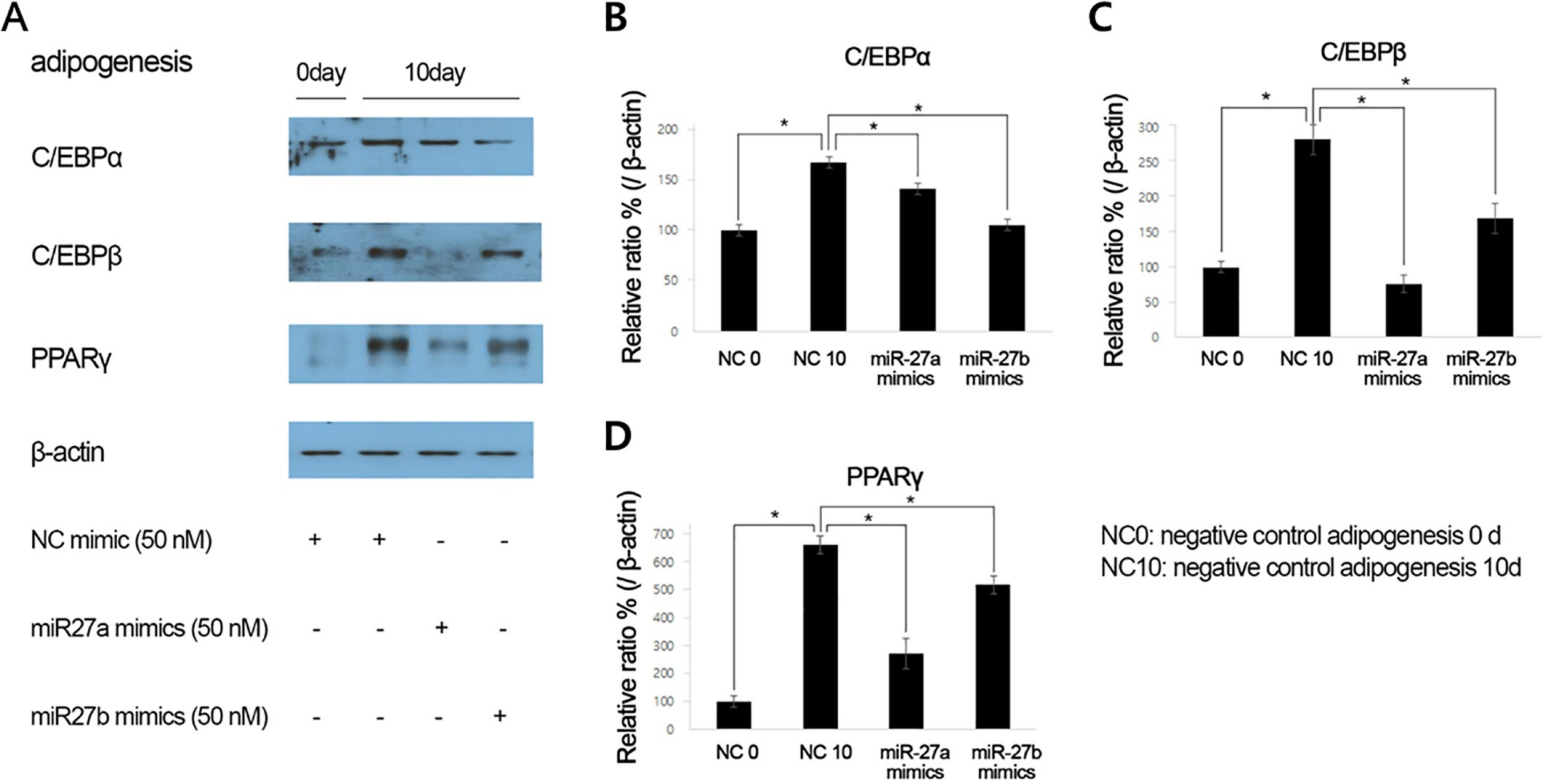
Effects of microRNA-27 (miR-27)a and miR-27b mimics on peroxisome-proliferator activated receptor-γ (PPARγ), CCAAT/enhancer binding protein (C/EBP)α, and C/EBPβ protein release in Graves’ orbitopathy orbital fibroblasts. (A) Representative photo of western blot analysis. (B) Protein levels of C/EBPα, (C) C/EBPβ and (D) PPARγ were increased on 10 day of adipogenesis and significantly decreased in orbital fibroblasts that were transfected with miR-27a and miR-27b mimics (*P<0.05). NC, negative control.

To confirm these findings in terms of protein expression, western blot analysis was performed; the results showed that the levels of adipogenic proteins including PPARγ, C/EBPα, and C/EBPβ, were decreased in GO orbital fibroblasts that were transfected with miR-27a and miR-27b mimics (Fig 5).

**Fig 6:**
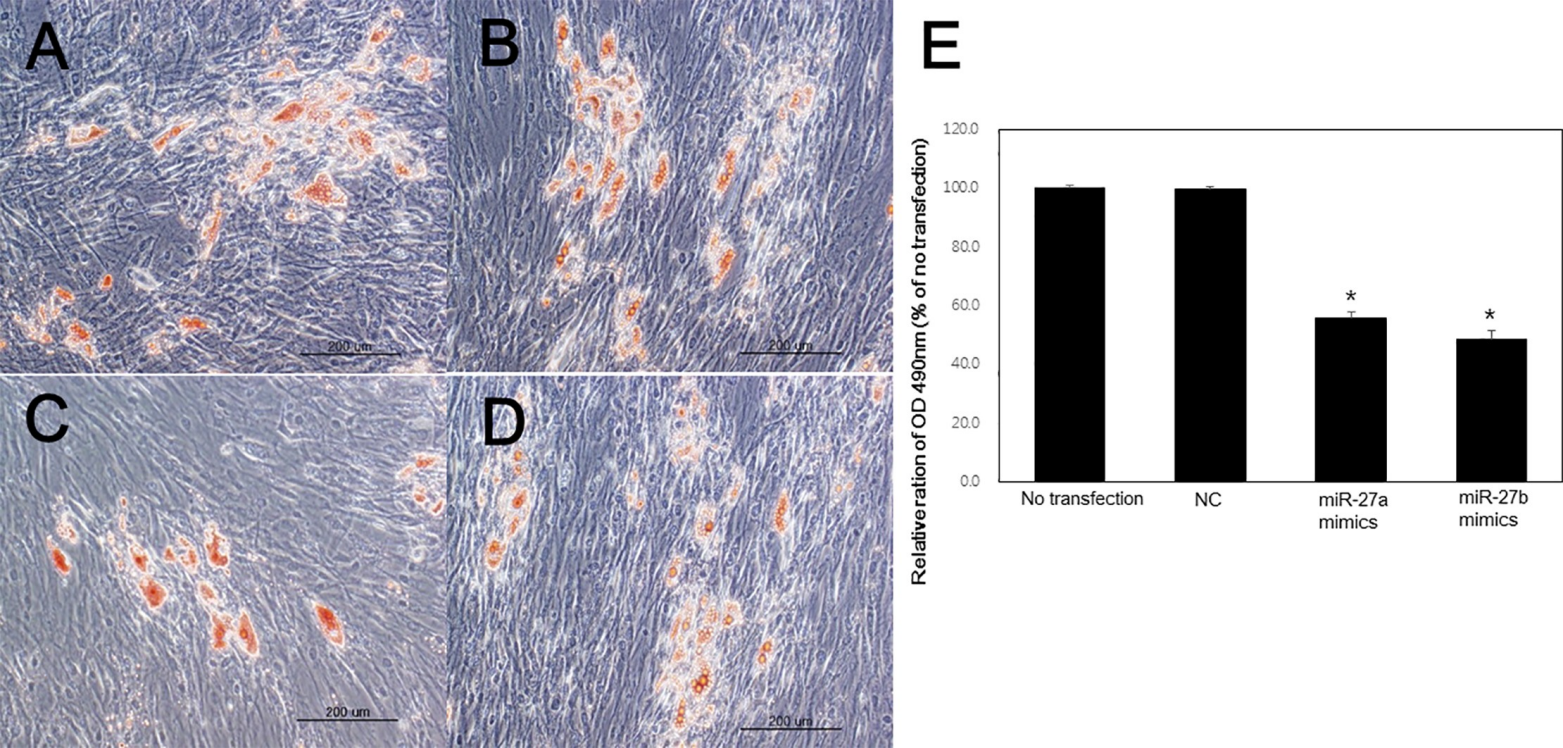
Oil red O staining after adipogenesis on day 10. (A) No transfection, (B) negative control mimics, (C) microRNA-27(miR-27)a-transfected orbital fibroblasts, (D) miR-27b-transfected orbital fibroblasts. Note that the levels of Oil Red O stained lipid droplets were reduced on day 10 in orbital fibroblasts that were transfected with the miR-27a or miR-27b mimic. (E) The optical density of miR-27a and miR-27b mimics transfected cell lysates showed significantly decreased absorbance at 490 nm (*P<0.01). NC, negative control.

The numbers of Oil Red O-stained lipid droplets were reduced at day 10 in GO fibroblasts that were transfected with miR-27a and miR-27b mimics (Fig 6). As results of quantification of Oil Red O staining, the optical density of miR-27a and miR-27b mimics transfected cell lysates showed significantly decreased absorbance at 490 nm compared to no transfected cell lysates (p < 0.01, respectively; independent *t*-test; Fig 6).

## Discussion

In the present study, we investigated the roles of miR-27a and miR-27b in adipogenesis in an *in vitro* model of GO. The results showed a significant decrease in miR-27a and miR-27b levels in orbital fat tissue from GO patients when compared to non-GO tissue. In GO orbital fibroblasts, the levels of miR-27a and miR-27b were gradually decreased upon adipogenic differentiation. Furthermore, the adipogenesis-induced increases of PPARγ, C/EBPα, and C/EBPβ proteins and mRNA were decreased in miR-27a and miR-27b mimic-transfected orbital fibroblasts. These results suggest that suppression of miR-27a and miR-27b might be involved in adipocyte development by repressing the expression of PPARγ and C/EBP.

Dysregulation of miRs has been found to be involved in various human diseases, such as cancer, inflammation, infection, and metabolic disorders [21]. Interest in adipogenesis has been increasing due to its relevance to obesity. MiRs can accelerate as well as suppress adipocyte differentiation [22]. For example, miR-143 is a miR that accelerates adipocyte differentiation [23,24]. Esau et al. [24] found that miR-143 levels increased in differentiating adipocytes, and inhibition of miR-143 inhibited adipocyte differentiation. In contrast, miR-27a and miR-27b impair adipocyte differentiation by targeting PPARγ [25,26]. Kim et al. [26] reported similar findings in studies using mice 3T3-L1 cells, and Karbiener et al. [25] reported similar results using human multipotent adipose-derived stem cells. The results in the present study were consistent with these previous reports. The expression levels of miRs that suppress adipocyte differentiation have been shown to be lower in differentiating adipocytes [15,25,26]. Likewise, the levels of miR-27a and miR-27b decreased upon adipogenesis in orbital fibroblasts from GO patients. These results suggest that miR-27a and miR-27b downregulation is necessary for adipocyte hypertrophy.

Because miRs play diverse and important roles in almost all aspects of cell physiology, it can be complex to apply miRs to clinical uses. Nevertheless, there have been studies on the use of miRs for clinical purposes. Recently, Mu et al. [27] suggested a new therapeutic strategy of the combined use of miRNA and anticancer drugs to increase drug responses in liver and kidney cancer patients. Liver and kidney cancers are notorious for drug resistance, and the authors found that miR-27b is sometimes deleted in liver and kidney cancers. Notably, the authors reported that miR-27b promoted drug responses in liver and kidney cancer patients in an *in vivo* study [27]. MiRs including miR-27a and miR-27b may provide therapeutic targets to treat fat metabolism disorders by controlling fat cell development.

In the present study, we used orbital fat tissues and primary cultures of orbital fibroblasts harvested from GO and non-GO participants. We compared the expression levels of miR-27a and miR-27b between GO and non-GO orbital fat tissues and found that the levels of both were significantly lower in GO fat tissue than in non-GO fat tissue. We also confirmed that miR-27a and miR-27b inhibited adipogenic differentiation in orbital fibroblasts from patients with GO. However, further studies using an experimental animal model of GO [28] are required to examine the potential of miR-27a and miR-27b as targets in GO treatments, especially for patients with disfiguring proptosis.

PPARγ and members of the C/EBP family are key players in adipocyte differentiation. Almost all genes involved in lipid metabolism have PPARγ:retinoid X receptor target sites [29,30]. In the present study, we found that the protein and mRNA expression levels of PPARγ, C/EBPα, and C/EBPβ were decreased in orbital fibroblasts that were transfected with either miR-27a or miR-27b mimics. However, the extents of the decreases were slightly different between miR-27a and miR-27b transfected cells. The miR-27a mimic more strongly inhibited PPARγ protein and mRNA expression than the miR-27b mimic in orbital fibroblasts. These findings were also observed in Oil Red O-staining adipocytes.

In conclusion, we demonstrated that miR-27a and miR-27b inhibited adipogenic differentiation in orbital fibroblast from patients with GO. Our data further support a novel negative regulatory mechanism of PPARγ-mediated adipogenesis by miR-27a and miR-27b, and suggest that miR-27a and miR-27b represent potential therapeutic targets for proptosis due to GO.

## Supporting information

S1 File Raw data of Figs 1–6. (XLSX)
